# Adsorption process and mechanism of heavy metal ions by different components of cells, using yeast (*Pichia pastoris*) and Cu^2+^ as biosorption models

**DOI:** 10.1039/d0ra09744f

**Published:** 2021-05-11

**Authors:** Xinggang Chen, Zhuang Tian, Haina Cheng, Gang Xu, Hongbo Zhou

**Affiliations:** School of Minerals Processing and Bioengineering, Central South University Changsha 410083 Hunan China xgchencsuft@163.com 1689256864@qq.com zhouhb@csu.edu.cn 443284336@qq.com; Key Laboratory of Biometallurgy of Ministry of Education Changsha 410083 China; Hunan Flag Bio-Tech Co., Ltd Changsha Hunan 410083 China xugang55@hotmail.com

## Abstract

Microbial biomass has been recognized as an essential biosorbent to remove heavy metal ions, but the biosorption process and mechanism of different components of microbial cells have not been elucidated. In present study, *Pichia pastoris* X33 and Cu^2+^ was used as a biosorption model to reveal the biosorption process and mechanism of different components of microbial cells. For the biosorption of whole cells, the maximum removal efficiency was 41.1%, and the adsorption capacity was 6.2 mg g^−1^. TEM-EDX analysis proved the existence of Cu^2+^ on the cell surface and cytoplasm. The maximum Cu^2+^ removal efficiency of the cell wall, cell membrane and cytoplasm were 21.2%, 20.7% and 18.5%, respectively. The optimum pH of Cu^2+^ biosorption of the *P. pastoris* cell, cell wall, cell membrane and cytoplasm was 6. Moreover, the maximum adsorption capacity of the cell, cell wall, cell membrane and cytoplasm was 16.13, 11.53, 10.97 and 8.87 mg g^−1^, respectively. The maximum removal efficiencies of *P. pastoris* biomass treated with proteinase K and *P. pastoris* biomass treated with β-mannanase were 18.1% and 28.2%, respectively. The maximum removal efficiencies of mannan and glucan were 34% and 12%, respectively. The FTIR spectra showed that the amino group (N–H), hydroxyl (O–H), carbon oxygen bond (C–O), –CH, C–N and carbonyl group (C=O) of a ketone or aldehyde may interact with Cu^2+^. Thus, our work provides guidance for further understanding the effect of different cell components on biosorption.

## Abstract 2

The Cu^2+^ first bound to the outer mannan and finally entered the cytoplasm. During the whole adsorption process, the number of adsorption sites in the outer and middle cell walls was the largest, and then gradually decreased.

## Graphical abstract

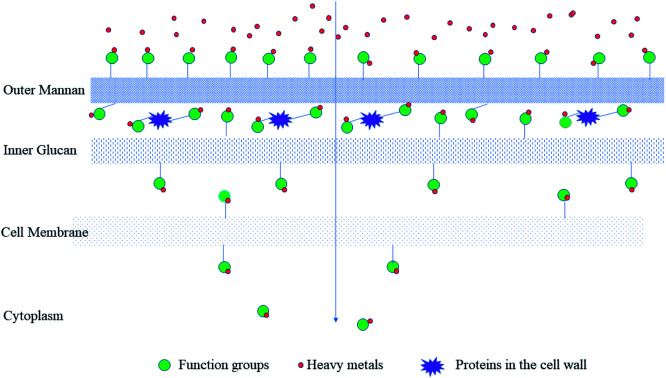

## 1. Introduction

With the development of industrialization, agricultural activities, and other human activity, various kinds of produced heavy metals have been discharged into water, water resources have been seriously polluted.^1,2^ Heavy metal contamination has been the focus for a long time due to its high toxicity and the difficulty of removal.^3–7^

Many efforts have been made to find a way to effectively reduce and remove heavy metals before waste water is discharged into the environment, such as ion exchange,^8^ chemical precipitation,^9^ evaporation, flotation, membrane filtration,^10^ electrochemical,^11^ coagulation–flocculation, and biosorption.^12^ Although these technologies have proven effective, the sustainability and economic cost was hard to be underestimated.^13^ Biosorption, which mainly used the organisms as adsorbents, may be an alternative owing to strong adaptability, low cost, no secondary pollution, low energy consumption and high efficiency. A large quantity of studies indicated that many organisms can efficiently adsorb heavy metal ions such as soybean meal waste, sugarcane bagasse, fungi and bacteria.^14^ However, most studies have only studied the biosorption properties of microbial intact cells, and limited reports have discussed the effects of microbial cell components on the biosorption process. Exploring the biosorption process and mechanism of different components of cells is very important for understanding biosorption.^15^

Element of Cu is a necessary element for biological growth. However, high concentrations of Cu are harmful to organisms.^16–19^ Excessive intake of Cu^2+^ leads to copper poisoning, diarrhea, epigastric pain, nausea and vomiting. Severe cases can lead to gastrointestinal mucosal ulcers, kidney damage, hemolysis, liver necrosis, shock, and even death.^20^ Therefore, the World Health Organization (WHO) have recommended copper concentration in drinking water not to exceed 1.3 mg L^−1^.^21^ However, copper is widely used in industrial productions such as electroplating, alloy manufacturing, refining processes and surface treatment industry, which inevitably resulted in the seriously pollution in water.^15,22–26^ Therefore, copper pollution is an urgent problem to be solved in heavy metal pollution, and the use of Cu^2+^ as the biosorption model of this study has certain representativeness.

Yeast, a traditional model fungus, is commonly used in genetic engineering and fermentation engineering.^27^ As a result, large quantities of waste yeast produced during these processes. Commonly, waste yeast biomass was used as organic fertilizer and feed.^28^ Recently, consideration of yeast as an inexpensive biosorbents for the removal of metal ions becomes the focus.^29^ The reason is there are many advantages of yeast, such as large-scale cultivation, low safety risks and easy to use.^15,30^ Many studies have studied the biosorption characteristics of yeast. Chen Can studied the morphological changes of *Saccharomyces cerevisiae* before and after biosorption of Ag^+^.^31^ M. Fadel explored the biosorption properties and optimum biosorption conditions of *Saccharomyces cerevisiae* for biosorption of manganese ions.^32^ Furthermore, Yunsong Zhang produced a bifunctional *Saccharomyces cerevisiae* as an adsorbent for Cd^2+^ or Pb^2+^ removal from aqueous solution.^33^ Fatemeh Elahian demonstrated that the genetically modified *Pichia pastoris* is a cost-effective, high-throughput, robust, and facile system for metal biosorption.^34^

All the above reports showed that yeast is an excellent and widely used adsorbent. However, the biosorption process is still unknown. *Pichia pastoris* was a typical model adsorbent, the exploration of the biosorption mechanism of different cell component can also provide a theoretical basis for understanding the biosorption mechanism of other adsorbents.

Here, *Pichia pastoris* X33 and Cu^2+^ were used as biosorption model. The biosorption process and mechanisms of *Pichia pastoris* were investigated. Firstly, through focusing on biosorption process of Cu^2+^ by *Pichia pastoris* biomass, the biosorption stage of Cu^2+^ by *Pichia pastoris* biomass was revealed. In addition, the relative biosorption ability of the main components of cell (cell wall, cell membrane and cytoplasm) and cell wall (glucan, protein, β-mannan) to Cu^2+^ was determined. Finally, the initial molecular mechanism of *Pichia pastoris* biosorption of Cu^2+^ was explored. Through this research we hope to provide a theoretical basis for the application of biological removal of heavy metals.

## 2. Materials and method

### 2.1 Preparation of solution

The copper sulphate pentahydrate was dissolved in deionized water to obtain a stock Cu^2+^ solution with a concentration of 400 mg L^−1^. The test solution with different concentrations was prepared by appropriately diluting the stock solution.

2 g L^−1^ dicyclohexanone oxalyldihydrazone (BCO) solution: 1 g BCO was heated and dissolved in 400 mL 50% ethanol solution (deionized water : ethanol = 1 : 1, V : V). After the solution was cooled, the volume was fixed to 500 mL.

pH 9.0 NH_4_Cl–NH_3_ buffer: 35 g of NH_4_Cl was dissolved in appropriate deionized water, and then 24 mL of 15 mol L^−1^ ammonia water was added. Finally, the volume of the solution was determined to 500 mL with deionized water.

500 g L^−1^ ammonium citrate solution: 250 g ammonium citrate was diluted to 500 mL with deionized water.

Proteinase K purchased from BioFroxx: enzyme activity ≥30 U mg^−1^, pH 6.2–6.8.

β-Mannanase purchased from Hunan Lerkam Biology Corp., Ltd: enzyme activity 50 000 U g^−1^, pH 5.0–6.0.

Snailase purchased from Sigma-Aldrich (Shanghai) Trading Co., Ltd: pH 5.2–7.2. Hypertonic buffer: 0.8 mol L^−1^ mannitol prepared by phosphate buffer (pH 6.0).

Pre-treatment agent: 0.1 g EDTA–Na_2_ and 0.1 mL β-mercaptoethanol was dissolved in 100 mL deionized water. 2% SDS buffer: 2 g sodium dodecyl sulfate (SDS) was dissolved in 100 mL deionized water.

pH 2.0–8.0 Na_2_HPO_4_–citric acid buffer: different volumes of 0.2 mol L^−1^ Na_2_HPO_4_ and 0.1 mol L^−1^ citric acid were dissolved to obtain different pH buffers. Different concentrations and pH of Cu^2+^ were prepared by dissolving copper sulphate pentahydrate in this buffer.

### 2.2 Preparation of biosorbents

*Pichia pastoris* X33 was acquired from Hunan Flag Bio-Tech Co., Ltd. It was preserved in Yeast Extract Peptone Dextrose (YPD) medium at 4 °C. Cells were cultivated in liquid YPD medium using a rotating incubator at 250 rpm and 30 °C for 12 h, the *P. pastoris* cells were harvested by centrifugation at 7000 rpm for 10 min. Each part of cells was obtained according to the following steps.

Cell wall: 0.5 g wet *P. pastoris* X33 biomass were suspended by 20 mL deionized water, frozen 1 h at −80 °C and thawed at room temperature. The above procedure was repeated 3 times to slightly break the *P. pastoris* cells. Then ultrasonic cell-break was carried out using a JY92-IIN ultrasonic cell breaker (SCIENTZ, China) under ice bath conditions. The cells were broken repeatedly until intact cells were failed to be identified by microscopy. Then, let the broken cell suspension centrifuged 10 000 rpm for 15 min.^35,36^ The broken cell suspension was then divided into two parts, the precipitation was the cell wall and the membrane, the supernatant was the cytoplasm. In order to remove the cell membrane mixed in the cell wall, the precipitation was extracted by boiling water bath with 20 mL 2% SDS buffer for 1 h and then washed with deionized water (10 000 rpm, 15 min) until the supernatant was clarified. The final precipitation was collected as the cell wall.^37,38^

Cell membrane and cytoplasm: 0.5 g wet *P. pastoris* X33 biomass were firstly suspended 30 min at 30 °C by 5 mL pre-treatment agent. Then, the 0.5 g wet *P. pastoris* X33 biomass which was treated by the pre-treatment agent were washed 2 times by hypertonic buffer (7000 rpm, 5 min). Next, 5 mL 2% snailase solution was added to the 0.5 g wet *P. pastoris* X33 biomass, and the mixture which contained *P. pastoris* X33 biomass and snailase was then oscillated for 6 h in the rotary shaker. After these steps, the mixture was washed with hypertonic buffer for 2 times to obtain protoplasts. 4 mL deionized water was added to the protoplasts to break the protoplasts. After that, the break protoplasts were centrifuged at 10000 rpm for 15 min. Consequently, the precipitation was the cell membrane, the supernatant was the cytoplasm.

*P. pastoris* treated with enzymes: 0.5 g wet *P. pastoris* X33 biomass were suspended 30 min at 30 °C by pre-treatment agent and then the 0.5 g wet *P. pastoris* X33 biomass which was treated by the pre-treatment agent were washed 2 times by hypertonic buffer (7000 rpm, 5 min), after that, the 0.5 g wet *P. pastoris* X33 biomass were treated 6 hours with 2% β-mannase and 2% protease K, respectively. Finally, *P. pastoris* biomass that treated with enzymes was harvested by centrifugation at 7000 rpm for 10 min.

### 2.3 Biosorption studies

Unless otherwise noted, the biosorption experiments were carried out with 20 mL system (20 mL of 100 mg L^−1^ Cu^2+^ and 0.5 g of wet biosorbents). Besides, the biosorption experiments were carried out on a mechanical shaker at 180 rpm, 25 °C. 1 mL samples were taken out at 5, 10, 15, 30, 60, 90, 120, 150 and 180 min. Samples were then centrifuged at 10 000 rpm for 5 min immediately to separate the biosorbents. After that, the supernatant was analyzed for residual Cu^2+^ concentration in the solution by spectrophotometry.

The procedure of spectrophotometric determination of Cu^2+^ concentration was as follows. Preparation of standard curve: transfer 0.0 mL, 0.2 mL, 0.4 mL, 0.8 mL, 1.2 mL, 1.6 mL, 2.0 mL and 2.4 mL of Cu^2+^ solution (10 mg L^−1^) into 8 colorimetric tubes, add 200 μL ammonium citrate solution, 500 μL NH_4_Cl–NH_3_ buffer, 500 μL BCO, and then add deionized water to 5 mL. Finally, the concentration of Cu^2+^ in the solution was analyzed using an Epoch UV-vis spectrophotometer (BioTek Inc, USA) at the wave length of 600 nm. The formula of standard curve was as follows:

1 *y* = 50.791*x* + 0.0559, *R*^2^ = 0.9992

where, *y* was the absorbance at 600 nm, *x* was the mass of Cu^2+^, mg. The Cu^2+^ concentration of the sample was also measured by the same method.

However, it is difficult to separate mannan, cytoplasm and glucan from Cu^2+^ solution by centrifugation. Therefore, the cytoplasm, glucan and mannan were injected into the dialysis bag respectively (Cu^2+^ could pass through the dialysis bag and the cytoplasm, glucan, mannan could not pass through the dialysis bag). Then put the dialysis bag into the 20 mL 100 mg mL^−1^ (final concentration) Cu^2+^ solution. Finally, the biosorption experiment was carried. The experiment of different pH on the biosorption was also the same as above, only the 100 mg g^−1^ Cu^2+^ was replaced with different pH 100 mg g^−1^ Cu^2+^. After 180 min, the concentration of Cu^2+^ was determined.

The Cu^2+^ biosorption capacity (*q*) (2) and removal efficiency (*R*) (1) were calculated through the following equations, respectively:2
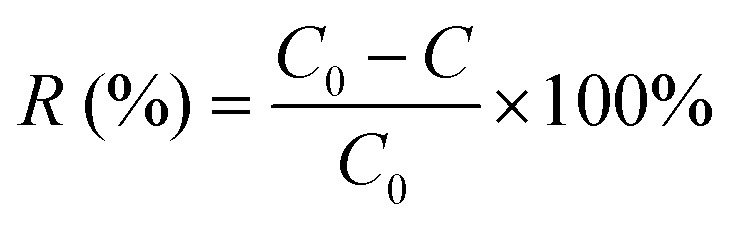
3
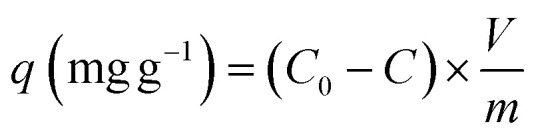
where, *q* (biosorption capacity) was the quantity of Cu^2+^ that be absorbed by the adsorbent per gram (mg g^−1^), *V* was the total volume of Cu^2+^ solution (L), *R* (removal efficiency) was proportion of Cu^2+^ adsorbed by *P. pastoris* to total Cu^2+^. *C* and *C*_0_ were the residual and initial concentrations of Cu^2+^, respectively. And *m* was the weight of biosorbents (g).

### 2.4 Isotherm studies

Biosorption equilibrium isotherms were performed in Erlenmeyer flasks with 0.5 g of dried biosorbents in 100 mL of a solution of Cu^2+^ at different concentrations (50 mg L^−1^, 100 mg L^−1^, 150 mg L^−1^, 200 mg L^−1^ and 250 mg L^−1^) and agitated at 180 rpm for 3 h in a shaker at 25 °C. Langmuir eqn (3) and Freundlich eqn (4) models were utilized to describe and evaluate the experimental data.^39,40^

Langmuir model:4
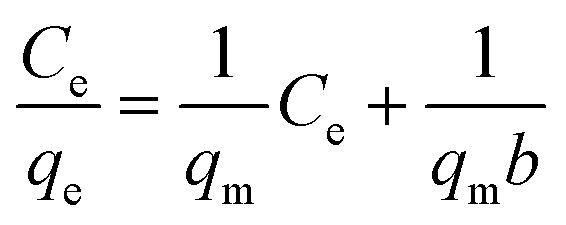


Freundlich model:5
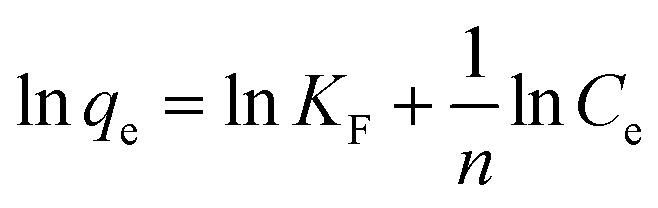
where, *q*_e_ was the equilibrium adsorption capacity of Cu^2+^ (mg g^−1^), *q*_m_ was the theoretical maximum adsorption capacity of Cu^2+^ (mg g^−1^), *C*_e_ was the Cu^2+^ concentration (mg L^−1^), *C*_0_ was the initial Cu^2+^ concentration (mg L^−1^), *b* was Langmuir constant (L mg^−1^); *K*_F_ was the Freundlich constant which represents the adsorption capacity, and *n* was the Freundlich equation constant.

### 2.5 Analytic methods

Transmission electron micrograph (TEM) and energy dispersive X-ray spectroscopy (EDX) experiments (TEM-EDX) were performed on a Tecnai G2 20S-Twin transmission electron microscope (FEI Czech Republic s. r. o, Czech) and a GENES XM60S Energy Dispersive Spectrometer (EDAX Inc, USA). The functional groups of the biosorbents were determined using a spectrum two Fourier transform infrared spectroscopy (PerkinElmer, UK). FTIR spectroscopy was carried out in the infrared region ranging from 4000 cm^−1^ to 450 cm^−1^. The spectra for before and after biosorption of the prepared adsorbents in the previous step were compared.

## 3. Results and discussion

### 3.1 Biosorption of Cu^2+^ by *P. pastoris* biomass

**Fig. 1:**
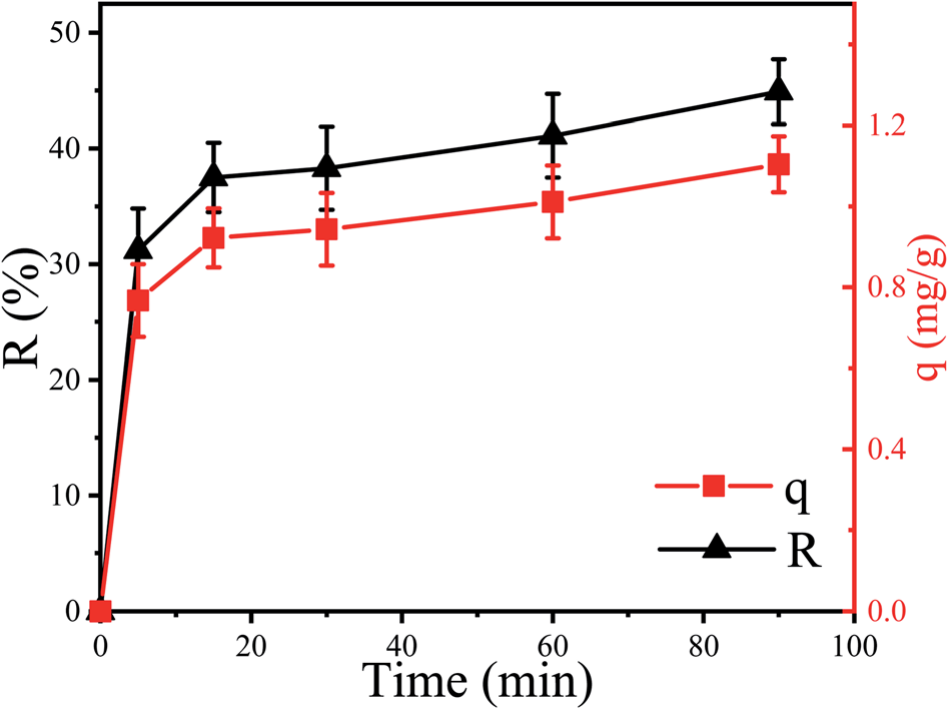
Biosorption of Cu^2+^ by *P. pastoris* biomass, initial Cu^2+^ concentration: 100 mg L^−1^, wet biomass dosage: 0.5 g, *R* – removal efficiency, *q* – biosorption capacity.

The removal efficiency (*R*) of Cu^2+^ by *P. pastoris* increased with increasing contact time (Fig. 1). In the first place, the removal percent of Cu^2+^ by *P. pastoris* rose sharply in first 5 min. After that, it rose slowly within 15 min. The biosorption equilibrium was established at 15 min and the maximum removal efficiency was 41.1%. However, no dramatic increase of removal efficiency was observed with a further extension of contact time (>15 min). Further observation showed that the maximum biosorption capacity (*q*) was 1.10 mg g^−1^ (6.2 mg g^−1^ for dry biomass), which was similar to the reported results (Fig. 1).^41^

Based on above results, it is suggested that there were two steps for Cu^2+^ biosorption: the first fast step lasted for 15 min (short time) and the second slow stage (long time) lasted until the equilibrium was established.^23,42^ It indicated that the biosorption of Cu^2+^ on the cell surface played an important role in the initial biosorption process. This was because a variety of macromolecules constitute the cell wall of fungi, including mannans, proteins, glucans, lipids, *etc.*^43^ These complex macromolecular structures provide potential binding sites for many different organic and inorganic molecules.^44^ To sum up, it was speculated that Cu^2+^ was firstly adsorbed on the cell surface and then slowly entered the cell.

### 3.2 TEM-EDX analysis

**Fig. 2:**
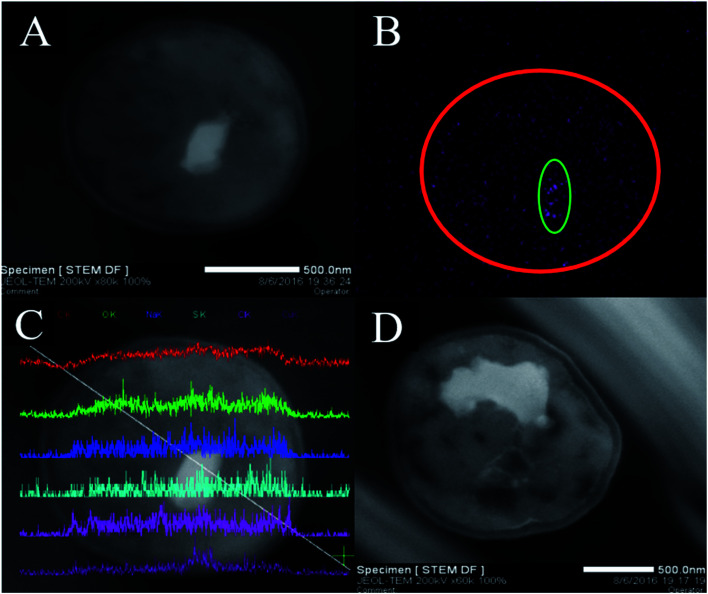
TEM-EDX analyses of Cu^2+^-loaded *P. pastoris* biomass after exposure to 100 mg L^−1^ Cu^2+^ for 1500 min, (A) TEM images of thin section of Cu^2+^-loaded *P. pastoris* cell; (B) elemental mapping of Cu^2+^-loaded *P. pastoris* cell, (C) line scan of Cu^2+^-loaded *P. pastoris* cell; (D) TEM images of thin section of Cu^2+^-loaded *P. pastoris* biomass.

To confirm that Cu^2+^ was firstly adsorbed on the cell surface and then slowly entered the cell, TEM-EDX experiments were performed. The TEM observation revealed that white irregular shaped precipitates (Cu^2+^) were accumulated in the cytoplasm (Fig. 2A and D). Additionally, the locations and shapes of these precipitates were different (Fig. 2A and D, which was in line with prior results.^45^ Therefore, we could preliminarily infer that Cu^2+^ entered the cell and distributed unevenly. Elemental mapping clearly revealed the distribution of Cu^2+^ in the cells (Fig. 2B). The purple dot corresponded to Cu^2+^ formed the shape of a cell (red circle) and was correlated with the brightness of Fig. 2A. In addition, the number of purple dots in the green circle increases significantly. The results indicated that Cu^2+^ was exactly entered into the *P. pastoris* cell.

TEM-EDX further confirmed that Cu^2+^ was adsorbed on the cell surface and inside. Six curves in Fig. 2C represented the abundance of C, O, Na, S, Cl, Cu elements from top to bottom. When the white line passed through the cell from the upper left to the lower right, the curves changed from left to right representing the change of element abundance. It could be seen that the white line entered the cell, the abundance of Cu^2+^ increased slightly. Then, the white line continued downward to the right, and entered the white precipitate. At this time, the abundance of Cu^2+^ was greatly improved. Moreover, when white lines passed through the white precipitates, the abundance of Cu^2+^ decreased dramatically. Finally, the white line passed through the cell, the abundance of Cu^2+^ decreased. Above evidences indicated that Cu^2+^ existed in the interior and surface of *Pichia pastoris* cell. Previous studies based on scanning electron microscopy (SEM) and EDX analysis also indicated that heavy metal ions could be adsorbed on the surface of cells.^46^

Therefore, biosorption and TEM-EDX experiments further explained that biosorption was carried out in two steps. Firstly, Cu^2+^ bound to functional groups on cell surface by ion-exchange or electrostatic interaction.^47,48^ Subsequently, Cu^2+^ passed through different layers of the cell wall (glucan, mannan and protein), then transported into the cell membrane, and eventually binds to the cytoplasm.

### 3.3 Biosorption of Cu^2+^ by *P. pastoris* cell components

**Fig. 3:**
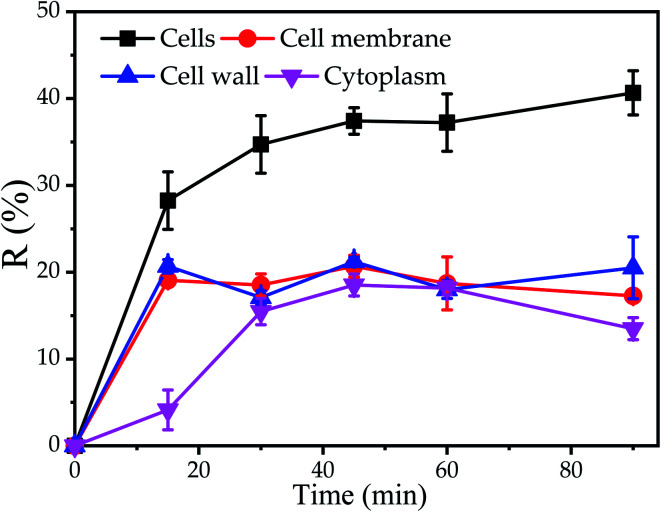
Biosorption of Cu^2+^ by *P. pastoris* cell components, initial Cu^2+^ concentration: 100 mg L^−1^, wet biomass dosage: 0.5 g, *R* – removal efficiency.

The results of TEM-EDX showed that Cu^2+^ could be adsorbed on cell surface and cytoplasm. Nevertheless, the influence of the different components of the cell on the biosorption was not clear. Consequently, the biosorption experiments of different components of cells were carried out. It was observed that the maximum Cu^2+^ removal efficiency of cell wall, cell membrane and cytoplasm were 21.2%, 20.7% and 18.5% at 45 min, indicated that the maximum removal efficiency of cell wall was found to be slightly higher as compared to cell membrane and cytoplasm, respectively. Furthermore, it was found that the equilibrium time of the cell and cytoplasm was 30 min, while the equilibrium time of the cell wall and cell membrane was 15 min, which further proved that the cell wall and cell membrane on the cell surface have a significant impact on the initial stage of biosorption (Fig. 3). The reason was supposed that cell disruption increased the contact area with Cu^2+^, cytoplasm, cell wall and cell membrane were not arranged neatly, so that the cytoplasm was exposed to the outside. E. Lopez Errasquin also believed that degraded cells, due to the destruction of cell membranes, provide more surface binding sites, greater available surface areas and expose intracellular components.^49^ However, the maximum removal rate of Cu^2+^ by cell wall, cell membrane and cytoplasm was lower than that of cell. The reason for this phenomenon may be that the cell components were prepared through a series of physical or chemical processes, such as ultrasound, Pre-treatment agent or SDS buffer, so that the cell components may lose some of the biosorption sites.

### 3.4 Effect of pH on biosorption of Cu^2+^ by *P. pastoris* cell components

**Fig. 4:**
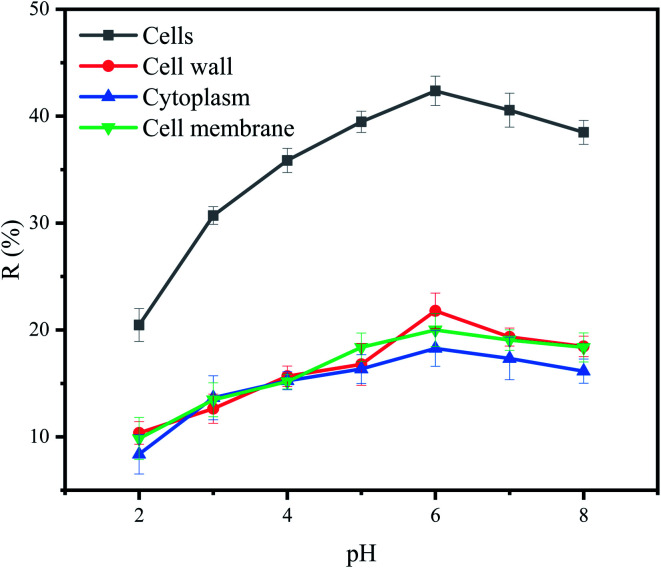
Effect of pH on removal of Cu^2+^ by *P. pastoris* cell components, initial Cu^2+^ concentration: 100 mg L^−1^, wet biomass dosage: 0.5 g, *R* Removal efficiency, initial Cu^2+^ concentration: 100 mg L^−1^, wet biomass dosage: 0.5 g, *R* – removal efficiency.

The effect of pH on removal of Cu^2+^ by each part of cells was shown in Fig. 4. Under different pH, the removal efficiency of Cu^2+^ by cells was relatively higher than that of other cell components. However, the effects of pH on cells and cell components were roughly the same. At lower pH, the amount of biosorption to Cu^2+^ was small. Biosorption to Cu^2+^ increased with the increased of pH from 2.0 to 6.0. The highest removal efficiency was observed in the pH 6.0. These observations can be explained by the fact that at lower pH values, the surface charge of the biomass is positive, which is not favorable to cations biosorption. Meanwhile, hydrogen ions compete strongly with Cu^2+^ at the active sites, resulting in less biosorption. With increasing pH, electrostatic repulsions between cations and surface sites and the competing effect of hydrogen ions decrease. Consequently, the metal biosorption increases.^50^ When pH was higher than 6, the OH^−^ competed with functional groups on the biosorbents to combine with Cu^2+^. It precipitates, and it will no longer be able to bond to the functional groups present in or on the biosorbents, leading to a decrease in its biosorption capacity as observed in this study.^51^

### 3.5 Biosorption isotherms of *P. pastoris* cell components

**Fig. 5:**
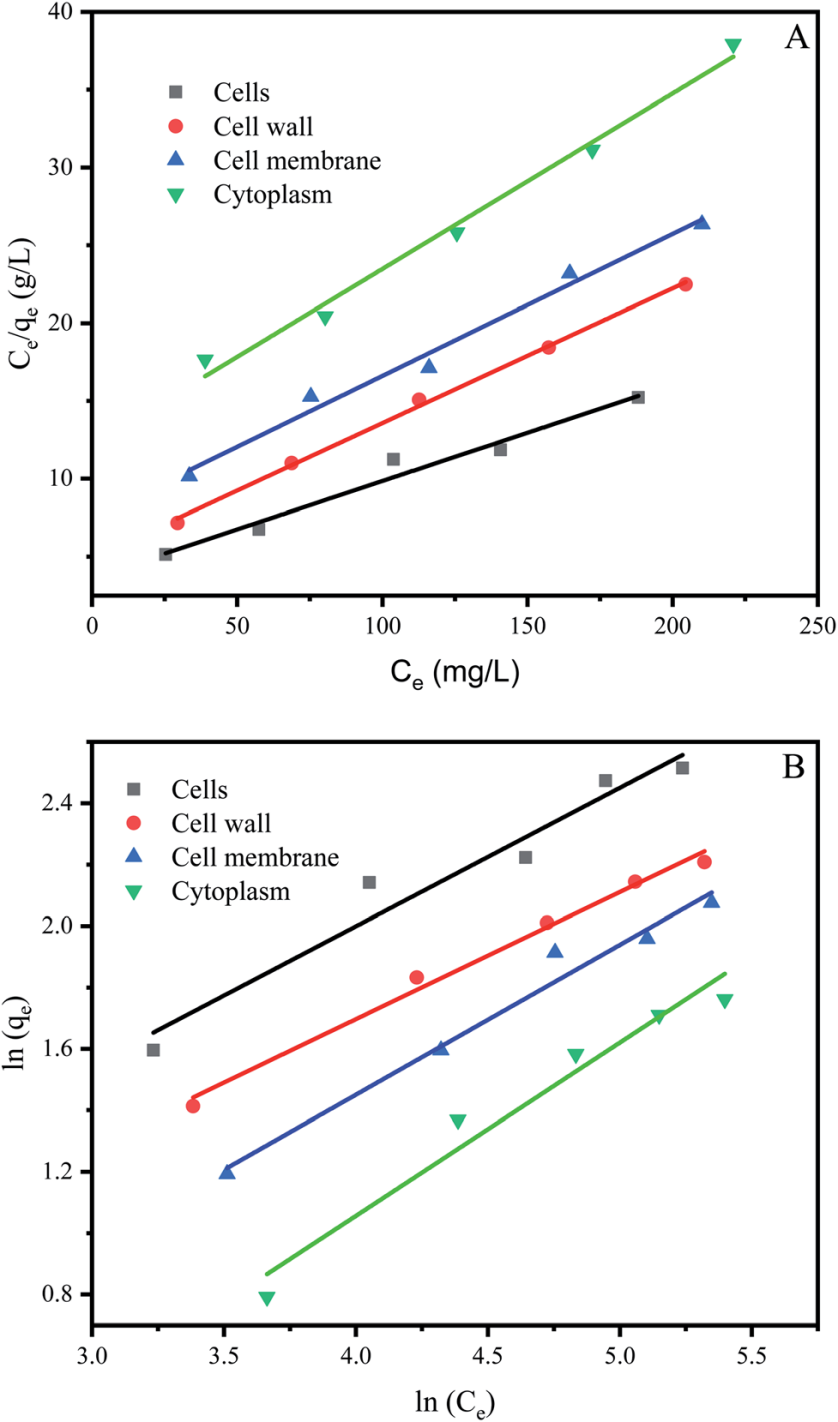
Biosorption isotherms of *P. pastoris* cell components. (A) Langmuir biosorption isotherm of Cu^2+^ with *P. pastoris* cell components, (B) Freundlich biosorption isotherm of Cu^2+^ with *P. pastoris* cell components, initial Cu^2+^ concentration: 100 mg L^−1^, wet biomass dosage: 0.5 g, *R* – removal efficiency.

The study of biosorption isotherm is of great significance in wastewater treatment as it provides valuable information for the pathway of biosorption reaction. In order to further explore the biosorption capacity of each cell component, the biosorption isotherm experiment was carried out. Fig. 5 showed the biosorption isotherms for intact cells, cell wall, membrane and cytoplasm. All the curve-fitting parameters were summarized in Table 1. A high correlation coefficient (*R*^2^ = 0.99) indicated that Langmuir model could better fit the biosorption process of cells, cell membranes, cells and cytoplasm, while both models could fit the biosorption process of cell wall. Other studies on biosorption by yeast also observed that the biosorption equilibrium isotherm was set to the model described by Langmuir.^51,52^

**Table 1:** Isotherm model parameters for biosorption of Cu^2+^ on *P. pastoris* cell components using Langmuir and Freundlich isotherms

	Langmuir model			Freundlich model		
Parameters	*q*_m_ (mg g^−1^)	*b* (L mg^−1^)	*R* ^2^	*K*_f_ (L g^−1^)	*n*	*R* ^2^
Cells	16.13	0.017	0.97	1.22	2.22	0.95
Cell wall	11.53	0.018	0.99	1.04	2.42	0.99
Cell membrane	10.97	0.012	0.99	0.61	2.05	0.98
Cytoplasm	8.87	0.009	0.99	0.30	1.77	0.96

The affinity constant *b* obtained from the Langmuir model was 0.017 for cells, 0.018 for cell wall, 0.012 for cell membrane and 0.009 for cytoplasm, respectively (Table 1). Thus, cell wall has a greater affinity for Cu^2+^ than other cell components. This may be because the cell wall has more biosorption sites than the cell membrane and cytoplasm, in addition the cell wall has more contact area than the cell. The maximum biosorption capacity (*q*_m_) of *Pichia pastoris* biomass was 16.13 mg g^−1^. The biosorption capacity of various other yeasts was measured as 2.59 to 76.8 mg g^−1^ for Cu^2+^ (Table 2). Table 2 indicated that most yeast with strong biosorption capacity have been modified. Among the unmodified yeast, *Pichia pastoris* used in this study has higher biosorption capacity, indicated that *Pichia pastoris* is a promising biosorbent. These results agreed with previous reports that the difference species and moisture content of yeast or initial concentration of Cu^2+^ significant affected the removal efficiency.^53^ However, the maximum adsorption capacity of cell, cell wall, cell membrane and cytoplasm were 16.13, 11.53, 10.97 and 8.87 mg g^−1^, respectively. Different from the rank of affinity constant *b*, the cell had the highest biosorption capacity, followed by cell wall, cell membrane and cytoplasm. This result was consistent with the result in Section 3.3, which showed that although the contact area between cells and Cu^2+^ was smaller than that of other cell components, the biosorption capacity of cells was still larger than that of other cell components due to the long contact time. This result also proved that the biosorption capacity of cell wall was higher than that of cell membrane and cytoplasm, which indicated that cell wall played an important role in the biosorption process.

**Table 2:** Biosorption parameter of Cu^2+^ on other yeasts biomass

Biosorbent	*q*_m_ (mg g^−1^)	pH	Equilibrium time	Ref.
Magnetically modified brewer's yeast	76.8	5–7	60 min	54
EDTAD-modified baker's yeast	65.0	6.0	20 min	55
baker's yeast treated with NaOH	27.79	5	20 min	56
Beer yeast	20.6	6	60 min	57
*S. cerevisiae* treated with NaOH	20	4.6	4 h	58
Heat pretreated baker's yeast	19.53	4.5	30 min	59
*Pichia stipitis*	16.94	4.5	10 d	60
*P. pastoris*	16.13	6.0	20 min	Present study
Baker's yeast treated with ethanol	15.64	5	20 min	56
*S. cerevisiae*	15.1	3.0	2 h	61
Baker's yeast	11.53	5	20 min	56
*S. cerevisiae* treated with ethanol	9.82	4.6	4 h	58
*S. cerevisiae*-Fe_3_O_4_	8.30	5.5	10 min × 9	62
*S. cerevisiae*	4.73	5	24 h	53
*S. cerevisiae*	4.70	5.5	10 min × 9	62
Baker's yeast	4.5	6.0	20 min	55
*S. cerevisiae*	2.59	3	1440 min	63

### 3.6 Effect of cell wall components of *Pichia pastoris* on biosorption of Cu^2+^

Above results indicated that the cell wall played an important role in the initial stage of biosorption, which may be related to some material in the cell wall. The dried cell wall of yeast approximately contains 13% protein and 59.8% polysaccharide, which consist of about 31% mannan, 28.8% glucan and 8.1% of lipids.^36,64^ In order to investigate the effect of protein and mannan in the cell wall on biosorption, the *P. pastoris* biomass was treated 6 hours with β-mannanase (50 U) and protease K (30 U), respectively, and then used for biosorption experiments.

**Fig. 6:**
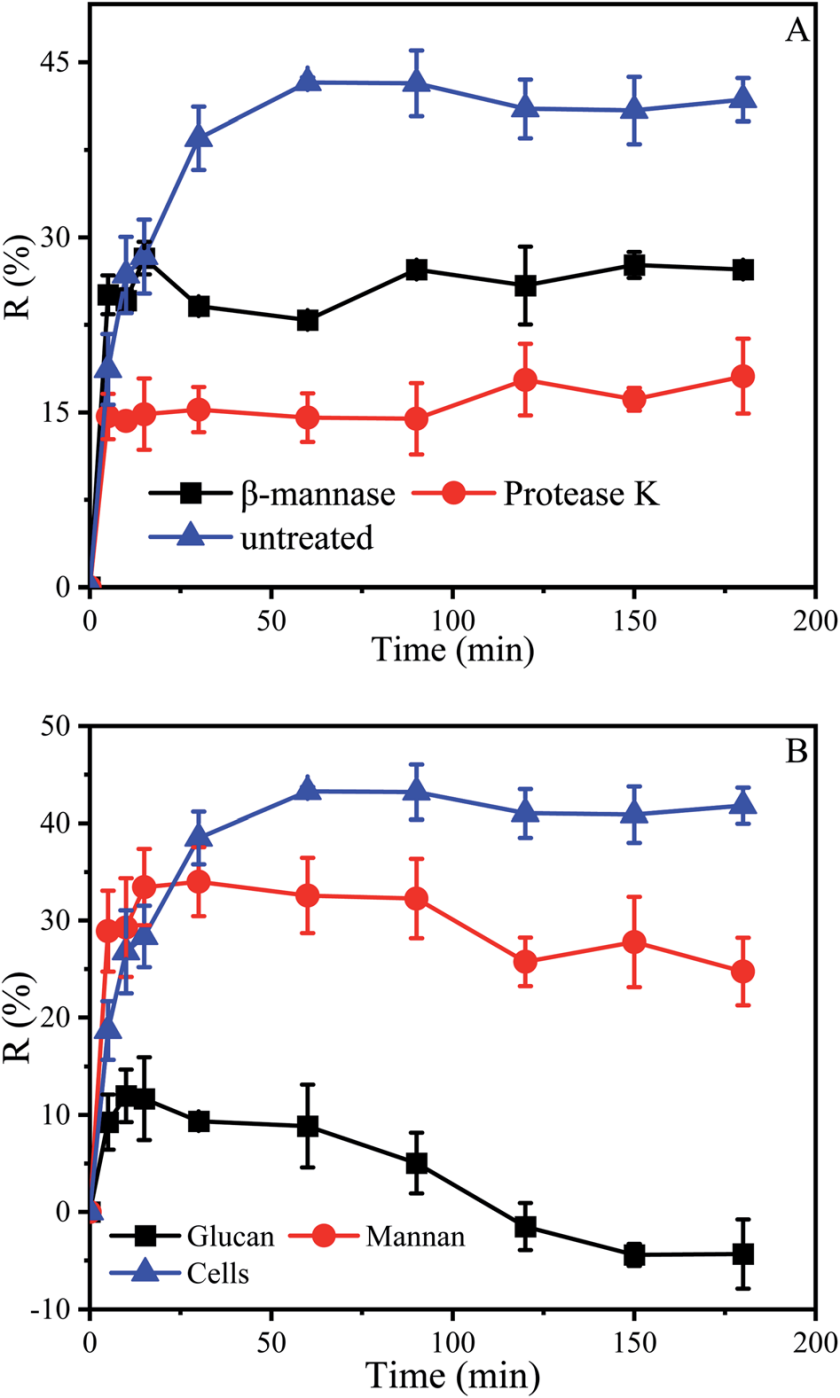
Effect of cell wall components of *Pichia pastoris* on biosorption of Cu^2+^. (A) Biosorption of Cu^2+^ by *P. pastoris* treated with enzymes, (B) biosorption of Cu^2+^ by glucan and mannan, initial Cu^2+^ concentration: 100 mg L^−1^, wet biomass dosage: 0.5 g, *R* – removal efficiency.

Results indicated that after treatment with protease K, the biosorption process had been greatly influenced. When the biosorption was finished, the maximum removal efficiencies of untreated *P. pastoris* biomass and treated with proteinase K were 43.2% and 18.1% respectively (Fig. 6A). The removal efficiency of *P. pastoris* biomass treated with proteinase K was only 41.9% of that in untreated *P. pastoris*. After treatment with β-mannanase, the removal efficiency was 28.2%, accounting for 65.3% of the removal efficiency of untreated *P. pastoris* biomass.

It proved that protein and mannan on the cell wall played an indispensable role in the biosorption of Cu^2+^. Previous studies also demonstrated that Cu^2+^ could be adsorbed by protein which directly influenced photosynthesis^65^ and caused oxidative damage of cells.^66^ Misbah Saleem explored the utilization of biomass from coconut copra meal (CCM) as a biosorbent to remove Ni^2+^ from aqueous solutions, and the main component of coconut powder is mannan, which also proved that mannan could adsorb heavy metal ions.^67^ Xuegang Luo also proved that the modified mannan had excellent biosorption properties for Cu^2+^.^68^

Not only that, the removal efficiency of Cu^2+^ by *Pichia pastoris* treated with protease K decreased more, which indicated that the protein had a greater impact on the entire biosorption process, even if the cell wall contained more mannans. The protein then appears to be the “glue” which affixes the two wall layers together, which results in the mannan-protein layer that may be almost completely removed by protease K, while the mannanase only removed some of the mannan and not the entire layer.^64^

As mentioned above, the polysaccharide content in the cell wall was 59.8%, of which glucan was as high as 31%. To further determine the relative biosorption capacity of glucan and mannan in the cell wall for Cu^2+^. Biosorption of Cu^2+^ by glucan and mannan was carried out.

Results indicated that the removal efficiency of mannan and *P. pastoris* reached 34% and 43.3%, respectively (Fig. 6B). However, the removal efficiency of Cu^2+^ by mannan was better than that of *P. pastoris* in the early stage of biosorption which may due to that the mannan on the cell wall is a three-dimensional arrangement. Therefore, at the first stage of biosorption, the groups on the cell surface combined with Cu^2+^. After that, the groups inside the cell functioned with the penetration of Cu^2+^. In contrast, all groups of the broken cell wall were exposed outside, Cu^2+^ can be fully contacted with the cell walls. The fact further indicated that mannan on *P. pastoris* surface also played an indispensable role in biosorption of Cu^2+^.

However, it was noticed that the role of glucan in the biosorption process was mediocre. When the reaction was carried out to 10 min, the removal efficiency of glucan was 12% and desorption occurred subsequently, agreed with prior results that desorption occurs in some environments (Fig. 6B).^69^ Removal efficiency is related to the properties of biosorbents, such as the amount of functional groups, molecular structure, swelling degree, surface area, and particle size.^70^ For instance, the basic unit of chitosan is glucosamine, which is similar to glucan in structure. However, in contrast to glucan, many reports have shown that chitosan has highly efficient adsorption of heavy metal ions.^71^ In addition, chitosan could be obtained by *N*-deacetylation of chitin contained in yeast cell wall. This is also the reason why many studies tend to modify yeast to improve its adsorption capacity (Table 2). Mannose and glucose are isomers, which results in mannan having some functions that glucan does not have. Therefore, mannan and glucan had essential differences in structure, which was the reason for their different removal efficiency.^72^ These results also suggested that we could modify the mannan in the cell wall to improve the adsorption capacity of yeast, and the modification of yeast was inseparable from the exploration of adsorption sites.

### 3.7 FTIR analysis

The FTIR spectrum could effectively identify functional groups that may bind to Cu^2+^.^73–75^ Each functional group has a specific absorption peak. When Cu^2+^ interacts with functional groups, the adsorption peaks of functional groups shift to higher or lower wave numbers.

**Fig. 7:**
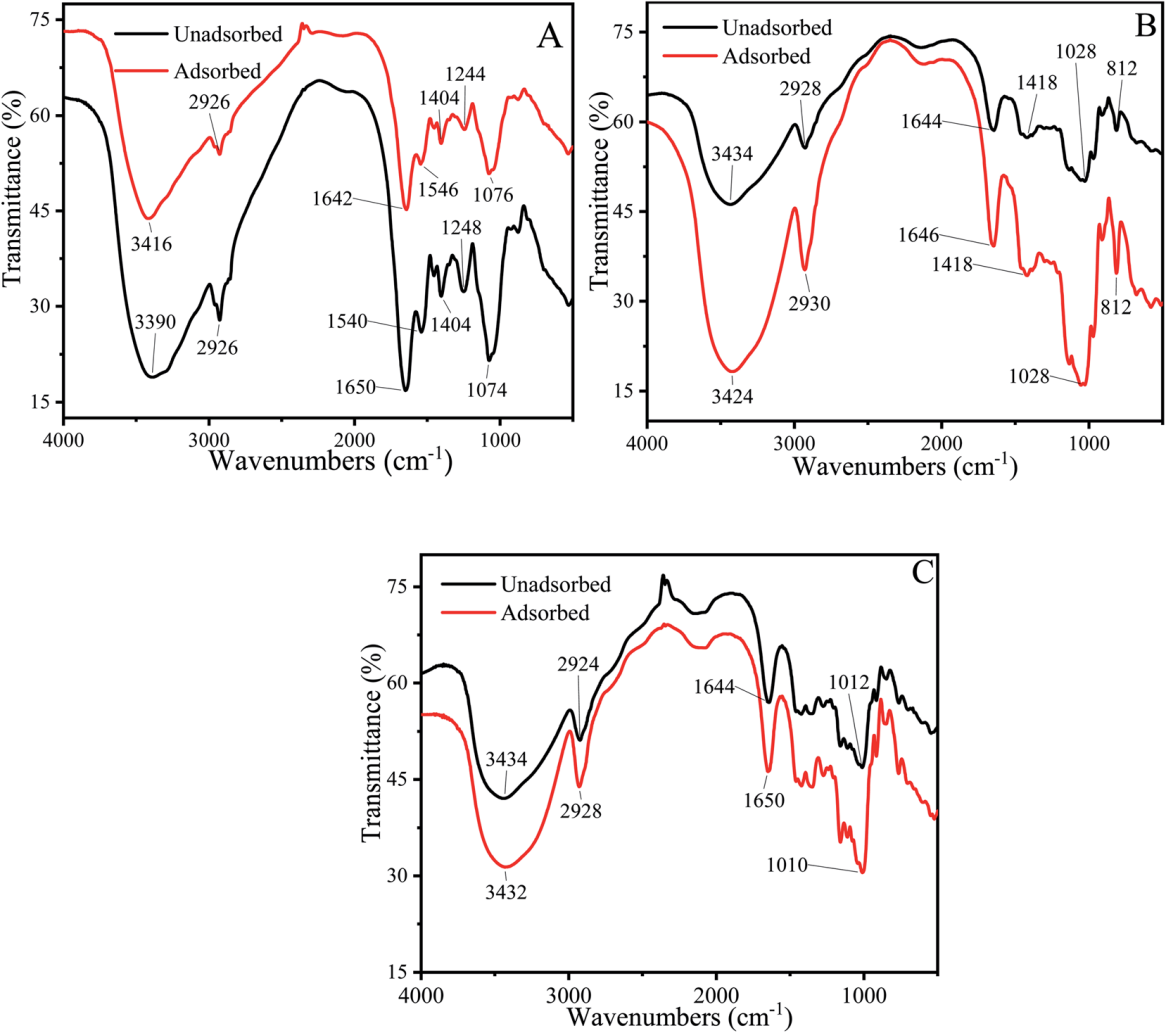
FTIR spectrum of unadsorbed and Cu^2+^-adsorbed biosorbents*.* (A) Cu^2+^-Adsorbed and unadsorbed *P. pastoris* biomass. (B) Cu^2+^-Adsorbed and unadsorbed mannan. (C) Cu^2+^-Adsorbed and unadsorbed glucan.

Fig. 7A described the FTIR spectrum of *P. pastoris* biomass. The broad and intensely stretched peak at 3390 cm^−1^ due to the presence of hydroxyl (O–H) stretching in hydrogen bonds. After the biosorption process, the peak shifted to 3416 cm^−1^, indicated that the hydroxyl from polysaccharides, fatty acids and protein were involved the biosorption of Cu^2+^.^76,77^ The peaks at 2926 cm^−1^ and 1404 cm^−1^ represented the asymmetric stretching vibration of –CH_2_ and bending vibration of –CH_3_ and –CH_2_. After the biosorption process, the peaks became more blunter, indicated that –CH_2_ and CH_3_ were the functional group to combine with Cu^2+^. Sławomir Wierzba also found that –CH_2_ and CH_3_ were the biosorption group in the yeast cell.^78^ The peaks observed at 1650 cm^−1^ was attributed to amide I band (stretching vibration of C=O), 1540 cm^−1^ indicated the amide II band (stretching vibration of C–N and bending vibration of N–H), 1248 cm^−1^ was attributed to amide III band (stretching vibration of C–N and bending vibration of N–H).^79,80^ After biosorption, the wavenumber shifted to 1642 cm^−1^,1546 cm^−1^,1244 cm^−1^ respectively, further proved that protein played an important role in the biosorption of Cu^2+^. The peak at 1074 cm^−1^ corresponded to stretching vibrations of C–O which from polysaccharides, after biosorption, the wavenumber shifted to1076 cm^−1^.^81^

Above results demonstrated that –OH, C–N, N–H, C=O, C–O and –CH from protein, polysaccharides and fatty acids were involved in Cu^2+^ biosorption process. It was consistent with previous biosorption experiment results. Haojie Huang also found that amide II, –CH_2_, hydroxyl group, acetylamino group and amide I on the surface of *E. coli* cells participated in the biosorption of heavy metals.^82^

To confirm the key groups of mannan and glucan to adsorb Cu^2+^, FTIR spectrum of mannan and glucan before and after biosorption were determined. Fig. 7B and C showed the groups whose peak shape changed significantly after mannan and glucan adsorbed Cu^2+^. Similar biosorption groups were present in glucan and mannan. However, the number of biosorption groups of mannan and glucan was significantly reduced.

Broad peak present in the 3434 cm^−1^ was attributed to the hydroxyl (–OH) stretching vibrations. After biosorption process, the wavenumber shifted to 3424 cm^−1^ and 3432 cm^−1^ respectively (Fig. 7B and C). It was suggested that the hydroxyl groups on mannan and glucan could also adsorb Cu^2+^, which was consistent with the previous experimental results. The peaks at 2928 cm^−1^ and 2924 cm^−1^ shifted to 2930 cm^−1^ and 2928 cm^−1^ respectively (Fig. 7B and C). The peak at 1418 cm^−1^ was also the –CH stretching vibration (Fig. 7B). After the biosorption process, the peak became acuter. It could be speculated that –CH_2_ and –CH_3_ in mannan and glucan were involved in biosorption of Cu^2+^. Similar results were found in other adsorbents.^77,83^ The peak observed at 1644 cm^−1^ was attributed to the C=O stretching vibration of mannan and glucan.^84^ After biosorption process, the wavenumbers shifted to 1646 cm^−1^ and 1650 cm^−1^ respectively, demonstrated that C=O in mannan and glucan could also bind to Cu^2+^. The peak at 1028 cm^−1^ was attributed to stretching vibration of C–O (Fig. 7B). After biosorption, the peak did not change, indicated that –C–O was not the main group to combine with Cu^2+^ in mannan (Fig. 7B). However, the peak at 1012 cm^−1^ shifted to 1010 cm^−1^ demonstrated that the C–O bond in glucan could bind to Cu^2+^ (Fig. 7C). The peak at 812 cm^−1^ was attributed to pyranose ring vibration band of mannose, which did not involve the biosorption of Cu^2+^ (Fig. 7B).^85^

Above results showed that the polysaccharides on the cell wall also had a certain number of biosorption groups, which further proved the importance of polysaccharides for biosorption (Fig. 7B and C). However, when there were only polysaccharides and no protein in the cell wall, the number of biosorption sites also decreased, which also indicated the importance of protein in biosorption (Fig. 7).

The FTIR spectra indicated that C=O, N–H, –OH, C–O, C–N, and –CH were responsible for biosorption of Cu^2+^. Although the biosorption sites of mannan and glucan decreased, their biosorption sites were consistent with *P. pastoris* biomass. Many studies have reported that these similar groups can adsorb heavy metal ions.^22,86–88^

## 4. Conclusions

Microorganism biomass could be used as an environmentally friendly and inexpensive adsorbent for heavy meatal biosorption from wastewater. However, the effect of each component of microbial cells on the adsorption of heavy metals is still unclear. In present study, *P. pastoris* and Cu^2+^ were used as models to explore the adsorption capacity of different microbial cell components for heavy metal ions. The biosorption experiment with 100 mg L^−1^ Cu^2+^ showed that the biosorption equilibrium time of *P. pastoris* biomass was 15 min, the maximum removal efficiency was 41.1%, and the adsorption capacity was 6.2 mg g^−1^. TEM-EDX showed that the associated Cu^2+^ heterogeneously present on the surface and inside of *P. pastoris* cells. These results indicated that the biosorption of Cu^2+^ on the cell surface played an important role in the initial biosorption process. The biosorption experiment with 100 mg L^−1^ Cu^2+^ indicated that the maximum Cu^2+^ removal efficiency of cell wall, cell membrane and cytoplasm were 21.2%, 20.7% and 18.5%, respectively. The optimum pH of Cu^2+^ biosorption on *P. pastoris* cell, cell wall, cell membrane and cytoplasm was 6. Moreover, the maximum adsorption capacity of cell, cell wall, cell membrane and cytoplasm were 16.13, 11.53, 10.97 and 8.87 mg g^−1^, respectively. Compared with other cell components, the adsorption capacity of cell wall was slightly higher, further indicated that cell wall played an important role in the initial biosorption process. In addition, the maximum removal efficiencies of *P. pastoris* biomass treated with proteinase K and *P. pastoris* biomass treated with β-mannanase were 18.1% and 28.2%, respectively, indicated that biosorption of Cu^2+^ was strongly influenced by the protein and mannan on the cell wall. The maximum removal efficiencies of mannan and glucan were 34% and 12%, respectively, indicated that the biosorption of mannan was stronger than that of glucan. Furthermore, the groups that could adsorb Cu^2+^ in *P. pastoris* biomass, mannan and glucan were hydroxyl (O–H), carbon oxygen bond (C–O), –CH, C–N and carbonyl group (C=O). In addition, amino group (N–H), and C–N in *P. pastoris* biomass could also adsorb Cu^2+^. Finally, this study proved that in the process of heavy metal biosorption, the cell wall has the strongest adsorption capacity, and the protein and mannan in the cell wall have great influence on the biosorption due to its rich adsorption sites. The results will provide a theoretical basis for further understanding of the mechanism of biosorption of heavy metal ions. In addition, the results can provide guidance for the modification of yeast to improve its adsorption capacity.

## Conflicts of interest

There are no conflicts to declare.

## Supplementary Material
